# Supplementary material for: Emergence of a Novel Genotype of Pigeon Deltacoronavirus Closely Related to Porcine Deltacoronavirus HKU15 and Sparrow Deltacoronavirus HKU17 in a Live Poultry Market in Shandong Province, China
Source: Microbiol Spectr. 2023 Jun 29;11(4):e00556-23. doi: 10.1128/spectrum.00556-23 (PMC10433798; doi:10.1128/spectrum.00556-23)

**Appendix**

**Methods** (pages 2-3)

**Table S1.** The prevalence of Gamma-CoV and Delta-CoV in samples collected from the live poultry market (page 4).

**Table S2.** Primers used for RT-PCR to confirm the NGS results of PiDCoV-WS38 and to obtain more sequence information of PiDCoV-WS31 (page 5).

**Table S3.** Comparison of nucleotide identity of complete genome (page 6).

**Table S4**. Comparison of amino acid identities between PiDCoV-WS38 and closely related Delta-CoVs (page 7).

**Table S5.** Comparison of amino acid identity of structural proteins (page 8).

**Table S6.** Comparison of amino acid identity of non-structural proteins (page 9).

**Table S7.** Accession numbers of the coronaviruses identified in this study (page 10-11).

**Figure S1.** The location of the live poultry market where the samples collected in Jining City, Shandong Province, China, 2022 (page 12).

**Figure S2.** Maximum-likelihood trees based on partial RdRp gene (page 13).

**Figure S3.** Maximum likelihood tree based on whole genome of coronaviruses (page 14).

**Figure S4**. Maximum-likelihood trees of ADRP, 3CL^pro^, RdRp, Hel, ExoN, NendoU, and O-MT replicase domains of coronaviruses (page 15).

**Figure S5.** Maximum-likelihood trees of non-structural 6 (NS6), non-structural 7a (NS7a), non-structural 7b (NS7b), and non-structural 7c (NS7c) proteins (page 16).

**Methods**

**RNA extraction and Pan-CoV RT-PCR**

Viral RNA was extracted from feces, swabs, and water fecal swabs of the live poultry market using QIAamp Viral RNA Mini Kits (Qiagen, No. 52904). Initial CoV screening was performed by a semi-nested PCR methods for the RNA-dependent RNA polymerase (RdRp) gene of CoVs (1). For first round RT-PCR, the external primers (pan-CoV_outF and pan-CoV_R) were used to amplify a 670–673 bp product; for the second round semi-nested PCR, an internal primer (pan-CoV_inF) was used with pan-CoV_R for amplification of an internal 599–602 bp product. To avoid possible contamination, negative controls were used in each step.

**Next-generation sequencing**

To get the whole genome of Delta-CoV strains detected by the Pan-CoV RT-PCR, the RNAs of two pigeon samples were subjected to next-generation sequencing (NGS). For library construction, RNAs were checked for integrity firstly by an Agilent 2100 Bioanalyzer (Agilent Technologies) and then rRNA was removed from each sample using NEBNext rRNA Depletion Kit (NEB), and the libraries were finally prepared using the NEBNext Ultra Ⅱ RNA Library Prep Kit for Illumina (NEB). Normalized library was sequenced on novaSeq 6000 platform (Illumina, San Diego, CA) using the 150 bp paired-end method.

**Bioinformatics analysis**

Raw sequence reads were filtered by removing adapters, low quality ends, repeated sequences, and ployX sequences using Soapnuke (v 2.0.5) for quality control (2). Then the host genome was removed from the clean reads using BWA (v 0.7.17) (3). Finally, the specific Delta-CoV reads were extracted and were de novo assembled with Megahit (v 1.1.2) (4).

**Phylogenetic analysis**

The sequences from the products of RT-PCR and NGS were deposited to GenBank (accession numbers OQ102159, OQ107215-107245, OQ938788-938820) (**Appendix Table 7**). The representative sequences from different genus of coronavirus were download from the GenBank database and the accession number of selected viruses can be found in **Appendix Figure S3**. The maximum-likelihood phylogenetic trees of CoVs were conducted using the Tamura-Nei model or Jones-Taylor-Thornton model in the software package Mega version X (University of Pittsburgh, Pittsburgh, PA). A bootstrap analysis was performed to assess the confidence limits of the branching with 1,000 replicates. Only values greater than 75 are included in the results.

**Reference**

1. Xiu L, Binder RA, Alarja NA, Kochek K, Coleman KK, Than ST, et al. A RT-PCR assay for the detection of coronaviruses from four genera. J Clin Virol. 2020 Jul;128:104391.

2. Chen Y, Chen Y, Shi C, Huang Z, Zhang Y, Li S, et al. SOAPnuke: a MapReduce acceleration-supported software for integrated quality control and preprocessing of high-throughput sequencing data. Gigascience. 2018 Jan 1;7(1):1-6.

3. Li H, Durbin R. Fast and accurate short read alignment with Burrows-Wheeler transform. Bioinformatics. 2009 Jul 15;25(14):1754-60.

4. Li D, Luo R, Liu CM, Leung CM, Ting HF, Sadakane K, et al. MEGAHIT v1.0: A fast and scalable metagenome assembler driven by advanced methodologies and community practices. Methods. 2016 Jun 1;102:3-11.

**Table S1.** The prevalence of Gamma-CoV and Delta-CoV in samples collected from the live poultry market.

| Sample Type | Total No. | Positive No. (%) | |
| --- | --- | --- | --- |
|  |  | Gamma-CoV | Delta-CoV |
| Feces | 20 | 11 (55.0) | 1 (5.0) |
| Board swab | 22 | 13 (59.1) | 0 (0) |
| Cage swab | 20 | 10 (50.0) | 0 (0) |
| Waste water | 22 | 14 (63.6) | 0 (0) |
| Drinking water | 22 | 13 (59.1) | 1 (4.5) |
| Total | 106 | 61 (57.5) | 2 (1.9) |

**Table S2.** Primers used for RT-PCR to confirm the NGS results of PiDCoV-WS38 and to obtain more sequence information of PiDCoV-WS31.

| Number | Primer name | Sequence (5'-3') | Amplicon length |
| --- | --- | --- | --- |
| 1 | WS38-19269F | CTATTCTTCGCTACCTCGTTG | 994bp |
|  | WS38-20242R | AGAGATGTTAAGCCAAACCTG |  |
| 2 | WS38-20008F | TACCTACCAGCCAATAGAGC | 988bp |
|  | WS38-20975R | CTTTTAATGTTGAGGTCTCCG |  |
| 3 | WS38-20896F | ACTAATGCCACCAATATGACC | 864bp |
|  | WS38-21739R | ATTAGCCACAGTGTTAAGAGC |  |
| 4 | WS38-21460F | TATTACAACGGCATCATGGTC | 997bp |
|  | WS38-22436R | TATTGTAGATGCCAACGTCCC |  |
| 5 | WS38-22085F | TTACACAAGCTGCACCCAA | 834bp |
|  | WS38-22898R | AATTTAACACAGGCTAGGCAA |  |
| 6 | WS38-22676F | CGATCTTTCTTTGCACTGGTT | 618bp |
|  | WS38-23274R | TGGCCCAAGAAATGAACGTA |  |
| 7 | WS38-23174F | TGCTCCAACCTTTCACCCTA | 935bp |
|  | WS38-24090R | GGAACTCCATTGCCACGAA |  |
| 8 | WS38-23892F | CCTTTGCTGCTTGAATATGGT | 644bp |
|  | WS38-24515R | TTTCCTTGGCTGATTATTGGT |  |
| 9 | WS38-24488F | TCTGGCTTCGGTAATAACTCC | 893bp |
|  | WS38-25360R | TAGTATAATCCTCGTGTCCCT |  |
| 10 | WS38-25054F | AATCTATGCCTGTCAAACCCA | 858bp |
|  | WS38-25892R | AATTTCCCCTAATTTGTCCC |  |

**Table S3**. Comparison of nucleotide identity of complete genome.

| Virus name | Host origin | PiDCoV-WS38 (%) |
| --- | --- | --- |
| SpDCoV-HKU17-6124 | Sparrow | 92.0 |
| SpDCoV-ISU690-4 | Sparrow | 86.7 |
| SpDCoV-ISU690-7 | Sparrow | 86.7 |
| SpDCoV-ISU42824 | Sparrow | 86.7 |
| SpDCoV-ISU73347 | Sparrow | 86.7 |
| HKU15-Haiti-Human-0081-4-2014 | Pig | 88.6 |
| HKU15-Haiti-Human-0329-4-2015 | Pig | 88.6 |
| HKU15-Haiti-Human-0256-1-2015 | Pig | 88.5 |
| HKU15-155 | Pig | 88.7 |
| HKU15-CHN-Tianjin-2016 | Pig | 88.6 |
| HKU15-USA-Arkansas-2015 | Pig | 88.6 |
| HKU11-934 | Bulbul | 72.9 |
| HKU12-600 | Thrush | 71.4 |
| HKU13-3514 | Munia | 75.3 |
| HKU16-6847 | White-eye | 71.5 |
| HKU18-chu3 | Magpie-robin | 74.5 |
| HKU19-6918 | Night-heron | 58.2 |
| HKU20-9243 | Wigeon | 57.2 |
| HKU21-8295 | Common moorhen | 66.4 |
| HKU27-988F | Falcon | 70.7 |
| HKU28-285F | Houbara | 70.7 |
| HKU29-271F | Pigeon | 70.7 |
| HKU30-411F | Quail | 84.5 |

**Table S4**. Comparison of amino acid identities between PiDCoV-WS38 and closely related Delta-CoVs.

| Domain | PiDCoV-WS38 (%) | | | |
| --- | --- | --- | --- | --- |
|  | SpDCoV-HKU17-6124 | SpDCoV-ISU690-4 | PDCoV-HKU15-155 | QuaCoV-HKU30-411F |
| ADRP | 100 | 95.8 | 96.6 | 92.4 |
| 3CLpro | 98.0 | 94.9 | 94.7 | 98.0 |
| RdRp | 99.6 | 97.3 | 97.8 | 95.9 |
| Hel | 100 | 99.1 | 99.4 | 99.1 |
| ExoN | 100 | 98.2 | 97.8 | 97.8 |
| NendoU | 98.0 | 91.8 | 95.2 | 90.5 |
| O-MT | 100 | 100 | 100 | 98.2 |
| Concatenated | 99.3 | 96.9 | 97.3 | 96.7 |

| **Table S5.** Comparison of amino acid identity of structural proteins. | | | | | |
| --- | --- | --- | --- | --- | --- |
| Virus name | PiDCoV-WS31/PiDCoV-WS38 | | | | |
|  | ORF1ab* | S | E | M | N |
| SpDCoV-HKU17-6124 | 99.1 | 49.7 | 94.0 | 94.9 | 99.2 |
| SpDCoV-ISU690-4 | 94.7 | 81.0 | 89.6 | 93.4 | 93.1 |
| SpDCoV-ISU690-7 | 94.7 | 81.0 | 89.6 | 93.4 | 93.1 |
| SpDCoV-ISU42824 | 94.6 | 85.3 | 91.0 | 92.9 | 92.3 |
| SpDCoV-ISU73347 | 94.6 | 85.3 | 91.0 | 93.4 | 92.3 |
| HKU15-Haiti-Human-0081-4-2014 | 95.6 | 88.0 | 92.5 | 93.4 | 96.4 |
| HKU15-Haiti-Human-0329-4-2015 | 95.6 | 88.1 | 92.5 | 93.4 | 96.4 |
| HKU15-Haiti-Human-0256-1-2015 | 95.7 | 88.3 | 92.5 | 93.4 | 96.8 |
| HKU15-155 | 95.9 | 87.9 | 92.5 | 92.9 | 97.2 |
| HKU15-CHN-Tianjin-2016 | 95.6 | 88.0 | 92.5 | 93.4 | 96.4 |
| HKU15-USA-Arkansas-2015 | 95.7 | 88.2 | 92.5 | 93.4 | 96.8 |
| HKU11-934 | 79.1 | 77.1 | 76.1 | 79.7 | 80.2 |
| HKU12-600 | 79.0 | 52.7 | 82.1 | 77.7 | 82.3 |
| HKU13-3514 | 82.8 | 77.0 | 76.1 | 74.6 | 82.7 |
| HKU16-6847 | 77.3 | 67.9 | 80.6 | 79.7 | 81.9 |
| HKU18-chu3 | 83.2 | 50.8 | 77.6 | 74.1 | 82.3 |
| HKU19-6918 | 55.6 | 46.9 | 41.8 | 55.8 | 56.5 |
| HKU20-9243 | 54.2 | 48.5 | 34.3 | 56.9 | 57.7 |
| HKU21-8295 | 72.8 | 58.0 | 67.2 | 64.5 | 67.3 |
| HKU27-988F | 78.2 | 49.5 | 82.1 | 80.7 | 83.9 |
| HKU28-285F | 78.1 | 49.8 | 82.1 | 80.7 | 83.5 |
| HKU29-271F | 78.1 | 49.8 | 82.1 | 80.7 | 83.5 |
| HKU30-411F | 92.8 | 78.7 | 92.5 | 89.3 | 91.9 |
| TCoV | 37.5 | 31.6 | 19.4 | 26.9 | 35.1 |
| IBV | 37.6 | 30.2 | 16.4 | 27.9 | 35.5 |
| IBV-partridge | 37.4 | 30.5 | 17.9 | 28.4 | 35.1 |
| SRAS-CoV-2 | 33.0 | 26.2 | 19.4 | 28.4 | 25.4 |
| SARS-CoV | 33.4 | 26.0 | 19.4 | 26.9 | 26.6 |
| MERS-CoV | 32.7 | 27.7 | 17.9 | 26.4 | 27.8 |
| Human-CoV-HKU1 | 31.9 | 27.0 | 19.4 | 25.4 | 32.3 |
| Human-CoV-OC43 | 32.3 | 26.7 | 14.9 | 28.4 | 28.6 |
| BCoV | 32.5 | 26.2 | 14.9 | 27.9 | 28.6 |
| PHEV | 32.5 | 26.9 | 14.9 | 26.9 | 28.6 |
| TGEV | 31.5 | 44.7 | 19.4 | 23.9 | 21.8 |
| Sc-BatCoV-512 | 31.2 | 47.2 | 11.9 | 23.9 | 21.0 |
| Human-CoV-NL63 | 31.3 | 45.6 | 10.4 | 20.8 | 19.0 |
| Human-CoV-229E | 31.6 | 46.4 | 17.9 | 24.9 | 23.0 |
| Canine-CoV-CCoV-HuPn-2018 | 31.5 | 44.2 | 20.9 | 23.4 | 21.4 |
| Canine-CoV-Z19 | 31.5 | 44.2 | 20.9 | 23.9 | 21.4 |
| PRCV | 31.4 | 45.0 | 19.4 | 23.9 | 21.8 |
| FIPV | 31.9 | 44.5 | 19.4 | 23.9 | 17.3 |
| PEDV | 31.3 | 47.1 | 14.9 | 23.4 | 20.6 |

*ORF1ab is only for PiDCoV-WS38.

**Table S6.** Comparison of amino acid identity of non-structural proteins.

| Virus name | PiDCoV-WS31/38 | | | |
| --- | --- | --- | --- | --- |
|  | NS6 | NS7a | NS7b | NS7c |
| SpDCoV-HKU17-6124 | 97.4 | 95.9 | 100.0 | 97.7 |
| SpDCoV-ISU690-4 | 94.8 | 91.8 | 69.0 | 63.6 |
| SpDCoV-ISU690-7 | 94.8 | 91.8 | 69.0 | 63.6 |
| SpDCoV-ISU42824 | 92.2 | 89.8 | 69.0 | 75.0 |
| SpDCoV-ISU73347 | 94.8 | 85.7 | 69.0 | 70.5 |
| HKU15-Haiti-Human-0081-4-2014 | 84.4 | 93.9 | NA | NA |
| HKU15-Haiti-Human-0329-4-2015 | 84.4 | 93.9 | NA | NA |
| HKU15-Haiti-Human-0256-1-2015 | 85.7 | 91.8 | NA | NA |
| HKU15-155 | 85.7 | 95.9 | NA | NA |
| HKU15-CHN-Tianjin-2016 | 84.4 | 93.9 | NA | NA |
| HKU15-USA-Arkansas-2015 | 85.7 | 91.8 | NA | NA |
| HKU11-934 | 62.3 | 6.1 | 14.3 | 36.4 |
| HKU12-600 | 70.1 | 6.1 | 21.4 | 36.4 |
| HKU13-3514 | 62.3 | 8.2 | 16.7 | 45.5 |
| HKU16-6847 | 72.7 | 53.1 | 14.3 | NA |
| HKU18-chu3 | 64.9 | 12.2 | 33.3 | 11.4 |
| HKU19-6918 | 29.9 | 12.2 | 2.4 | NA |
| HKU20-9243 | 44.2 | 14.3 | 4.8 | 6.8 |
| HKU21-8295 | 31.2 | 4.1 | 11.9 | 20.5 |
| HKU27-988F | 74.0 | 53.1 | 4.8 | 11.4 |
| HKU28-285F | 74.0 | 51.0 | 4.8 | 11.4 |
| HKU29-271F | 74.0 | 51.0 | 4.8 | 11.4 |
| HKU30-411F | 89.6 | 81.6 | 61.9 | 31.8 |

NA, not available.

**Table S7.** Accession numbers of the coronaviruses identified in this study.

| Virus name | Virus genus | Accession number | Gene |
| --- | --- | --- | --- |
| PigeonCoV-WS38 | *Deltacoronavirus* | OQ102159 | Complete genome |
| PigeonCoV-WS01 | *Gammacoronavirus* | OQ107215 | Partial RdRp gene |
| ChickenCoV-WS02 | *Gammacoronavirus* | OQ107216 | Partial RdRp gene |
| PigeonCoV-WS04 | *Gammacoronavirus* | OQ107217 | Partial RdRp gene |
| AvianCoV-WS06 | *Gammacoronavirus* | OQ107218 | Partial RdRp gene |
| AvianCoV-WS07 | *Gammacoronavirus* | OQ107219 | Partial RdRp gene |
| PigeonCoV-WS09 | *Gammacoronavirus* | OQ107220 | Partial RdRp gene |
| ChickenCoV-WS10 | *Gammacoronavirus* | OQ107221 | Partial RdRp gene |
| ChickenCoV-WS11 | *Gammacoronavirus* | OQ107222 | Partial RdRp gene |
| PigeonCoV-WS12 | *Gammacoronavirus* | OQ107223 | Partial RdRp gene |
| PigeonCoV-WS13 | *Gammacoronavirus* | OQ107224 | Partial RdRp gene |
| ChickenCoV-WS14 | *Gammacoronavirus* | OQ107225 | Partial RdRp gene |
| AvianCoV-WS15 | *Gammacoronavirus* | OQ107226 | Partial RdRp gene |
| AvianCoV-WS16 | *Gammacoronavirus* | OQ107227 | Partial RdRp gene |
| PigeonCoV-WS17 | *Gammacoronavirus* | OQ107228 | Partial RdRp gene |
| AvianCoV-WS20 | *Gammacoronavirus* | OQ107229 | Partial RdRp gene |
| ChickenCoV-WS22 | *Gammacoronavirus* | OQ107230 | Partial RdRp gene |
| PigeonCoV-WS27 | *Gammacoronavirus* | OQ107231 | Partial RdRp gene |
| ChickenCoV-WS28 | *Gammacoronavirus* | OQ107232 | Partial RdRp gene |
| AvianCoV-WS35 | *Gammacoronavirus* | OQ107233 | Partial RdRp gene |
| AvianCoV-WS36 | *Gammacoronavirus* | OQ107234 | Partial RdRp gene |
| ChickenCoV-WS37 | *Gammacoronavirus* | OQ107235 | Partial RdRp gene |
| AvianCoV-WS40 | *Gammacoronavirus* | OQ107236 | Partial RdRp gene |
| PigeonCoV-WS41 | *Gammacoronavirus* | OQ107237 | Partial RdRp gene |
| ChickenCoV-WS42 | *Gammacoronavirus* | OQ107238 | Partial RdRp gene |
| ChickenCoV-WS44 | *Gammacoronavirus* | OQ107239 | Partial RdRp gene |
| AvianCoV-WS48 | *Gammacoronavirus* | OQ107240 | Partial RdRp gene |
| AvianCoV-WS49 | *Gammacoronavirus* | OQ107241 | Partial RdRp gene |
| AvianCoV-WS50 | *Gammacoronavirus* | OQ107242 | Partial RdRp gene |
| PigeonCoV-WS31 | *Deltacoronavirus* | OQ107243 | Partial RdRp gene |
| PigeonCoV-WS38 | *Deltacoronavirus* | OQ107244 | Partial RdRp gene |
| PigeonCoV-WS31 | *Deltacoronavirus* | OQ107245 | Complete S_E_M_N_NS gene |
| ChickenCoV-WS51 | *Gammacoronavirus* | OQ938788 | Partial RdRp gene |
| ChickenCoV-WS53 | *Gammacoronavirus* | OQ938789 | Partial RdRp gene |
| PigeonCoV-WS54 | *Gammacoronavirus* | OQ938790 | Partial RdRp gene |
| PigeonCoV-WS55 | *Gammacoronavirus* | OQ938791 | Partial RdRp gene |
| PigeonCoV-WS56 | *Gammacoronavirus* | OQ938792 | Partial RdRp gene |
| PigeonCoV-WS57 | *Gammacoronavirus* | OQ938793 | Partial RdRp gene |
| PigeonCoV-WS58 | *Gammacoronavirus* | OQ938794 | Partial RdRp gene |
| PigeonCoV-WS59 | *Gammacoronavirus* | OQ938795 | Partial RdRp gene |
| PigeonCoV-WS60 | *Gammacoronavirus* | OQ938796 | Partial RdRp gene |
| AvianCoV-C22244 | *Gammacoronavirus* | OQ938797 | Partial RdRp gene |
| AvianCoV-C22245 | *Gammacoronavirus* | OQ938798 | Partial RdRp gene |
| AvianCoV-C22247 | *Gammacoronavirus* | OQ938799 | Partial RdRp gene |
| AvianCoV-C22248 | *Gammacoronavirus* | OQ938800 | Partial RdRp gene |
| AvianCoV-C22249 | *Gammacoronavirus* | OQ938801 | Partial RdRp gene |
| AvianCoV-C22250 | *Gammacoronavirus* | OQ938802 | Partial RdRp gene |
| AvianCoV-C22251 | *Gammacoronavirus* | OQ938803 | Partial RdRp gene |
| AvianCoV-C22252 | *Gammacoronavirus* | OQ938804 | Partial RdRp gene |
| AvianCoV-C22253 | *Gammacoronavirus* | OQ938805 | Partial RdRp gene |
| AvianCoV-C22262 | *Gammacoronavirus* | OQ938806 | Partial RdRp gene |
| AvianCoV-C22263 | *Gammacoronavirus* | OQ938807 | Partial RdRp gene |
| AvianCoV-C22266 | *Gammacoronavirus* | OQ938808 | Partial RdRp gene |
| AvianCoV-C22274 | *Gammacoronavirus* | OQ938809 | Partial RdRp gene |
| AvianCoV-C22275 | *Gammacoronavirus* | OQ938810 | Partial RdRp gene |
| AvianCoV-C22276 | *Gammacoronavirus* | OQ938811 | Partial RdRp gene |
| AvianCoV-C22277 | *Gammacoronavirus* | OQ938812 | Partial RdRp gene |
| AvianCoV-C22278 | *Gammacoronavirus* | OQ938813 | Partial RdRp gene |
| AvianCoV-C22279 | *Gammacoronavirus* | OQ938814 | Partial RdRp gene |
| AvianCoV-C22280 | *Gammacoronavirus* | OQ938815 | Partial RdRp gene |
| AvianCoV-C22281 | *Gammacoronavirus* | OQ938816 | Partial RdRp gene |
| AvianCoV-C22282 | *Gammacoronavirus* | OQ938817 | Partial RdRp gene |
| AvianCoV-C22283 | *Gammacoronavirus* | OQ938818 | Partial RdRp gene |
| AvianCoV-C23034 | *Gammacoronavirus* | OQ938819 | Partial RdRp gene |
| AvianCoV-C23040 | *Gammacoronavirus* | OQ938820 | Partial RdRp gene |

**Figure S1.** The location of the live poultry market where the samples collected in Jining City, Shandong Province, China, 2022-2023.

**
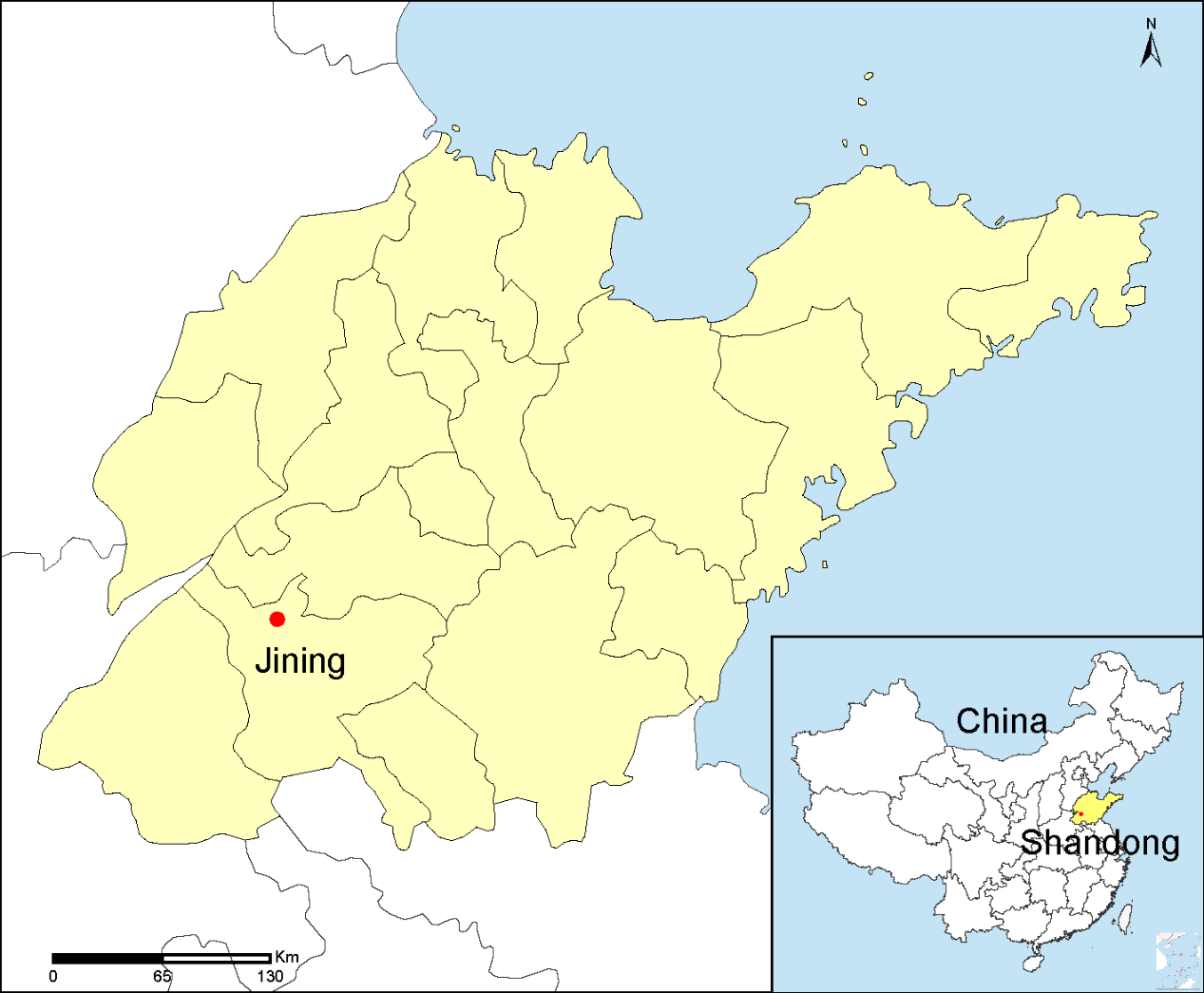
**

**Figure S2.** Maximum-likelihood trees based on partial RdRp gene of (A) Gamma-CoV and (B) Delta-CoV. The black dots represent the CoV strains identified in this study.


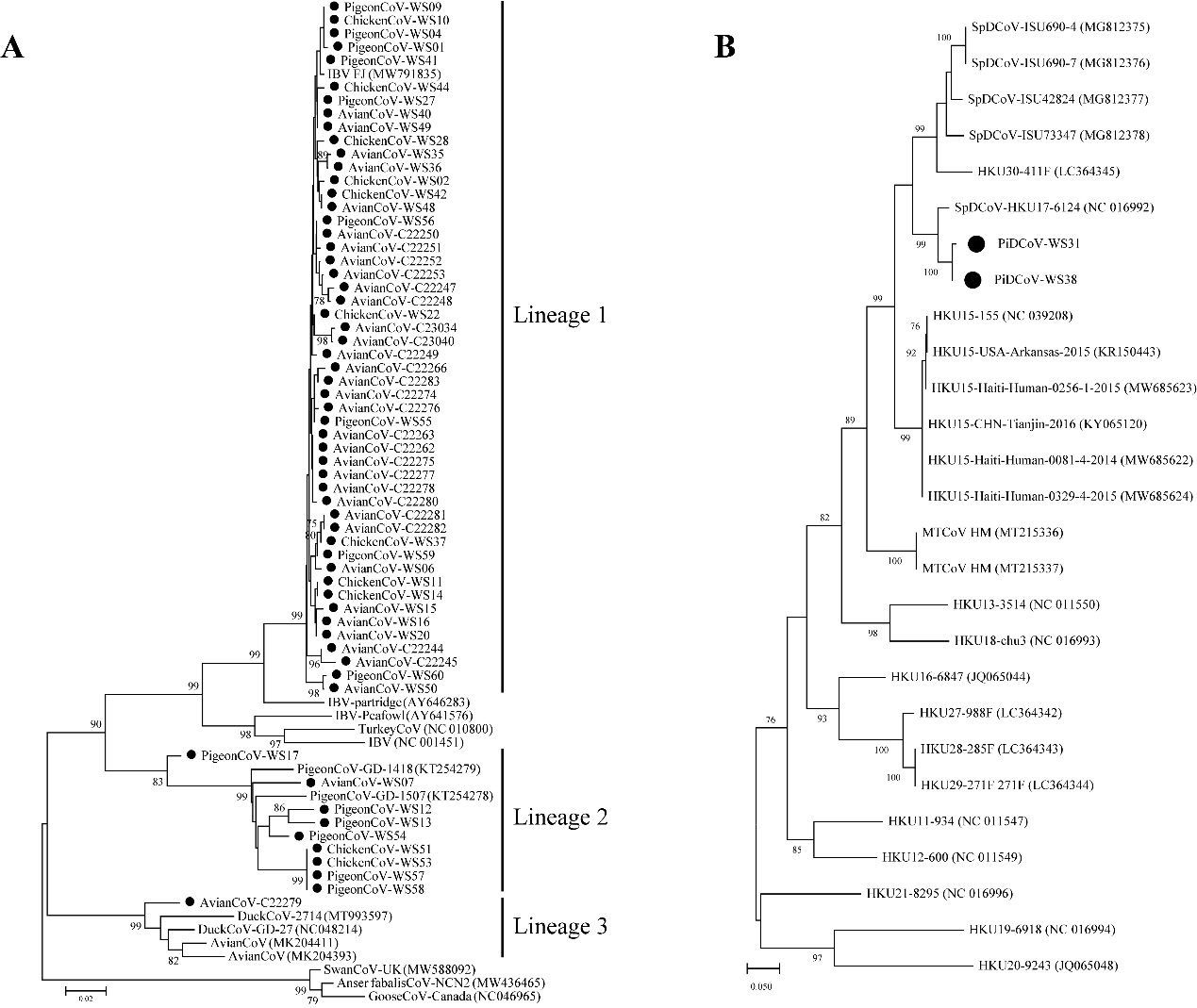


**Figure S3.** Maximum likelihood tree based on whole genome of coronaviruses. The red, blue, and orange dotes represent PiDCoV, SpDCoV, and PDCoV strains, respectively. The strains with red front represent the virus that can infect humans.


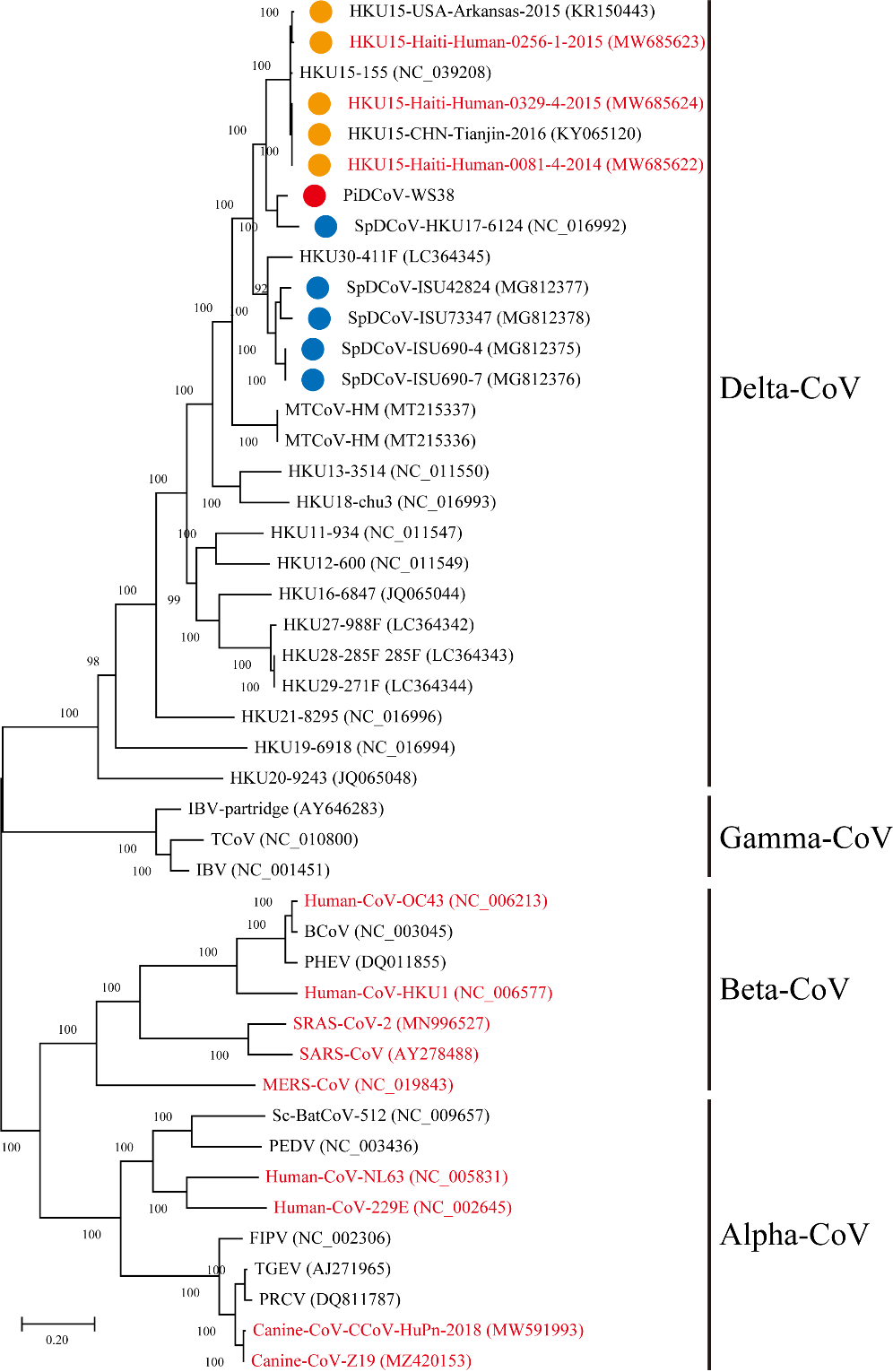


**Figure S4**. Maximum-likelihood trees of ADRP, 3CL^pro^, RdRp, Hel, ExoN, NendoU, and O-MT replicase domains of coronaviruses. The red, blue, and orange dotes represent PiDCoV, SpDCoV, and PDCoV strains, respectively. The strains with red front represent the virus that can infect humans.


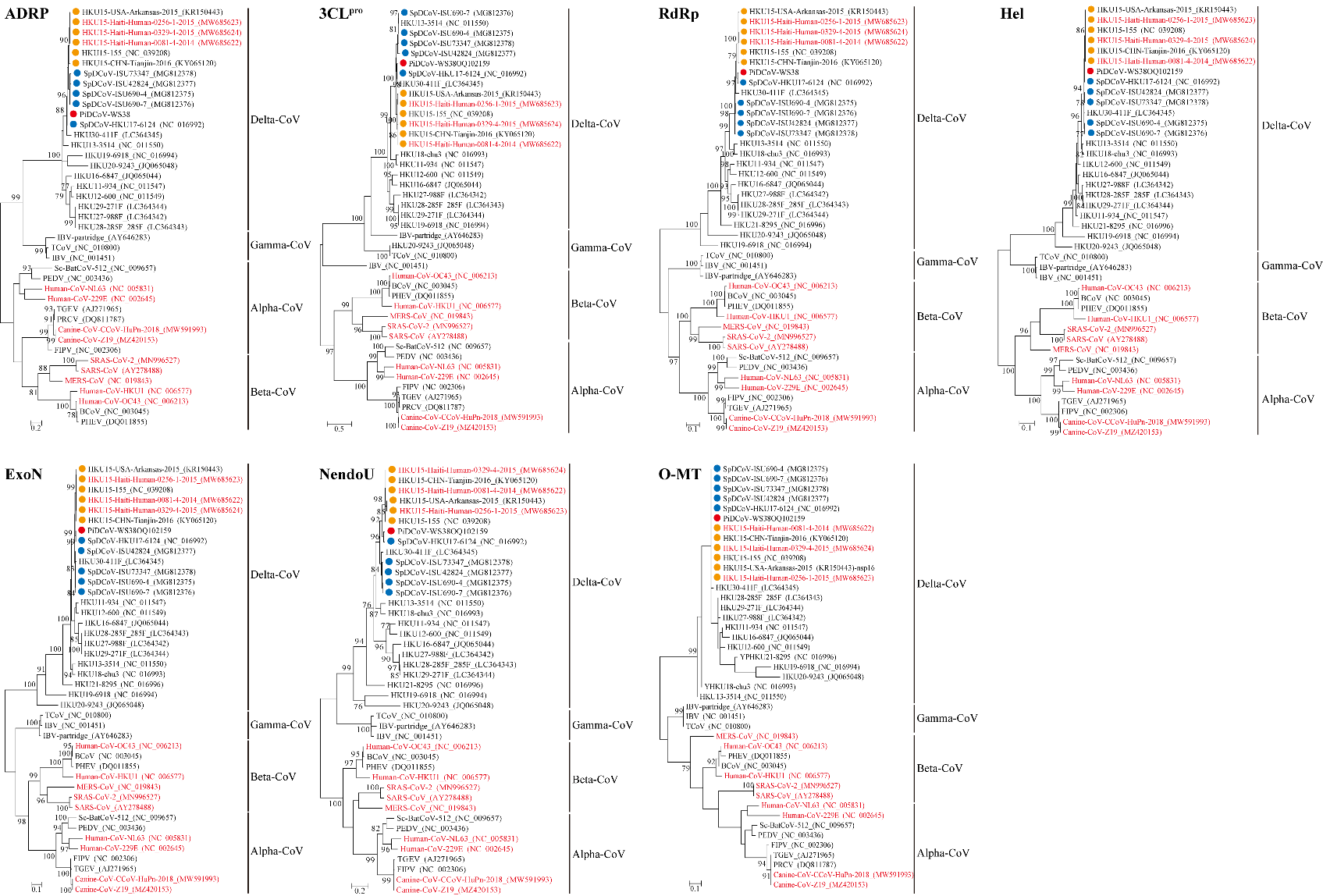


**Figure S5**. Maximum-likelihood trees of non-structural 6 (NS6), non-structural 7a (NS7a), non-structural 7b (NS7b), and non-structural 7c (NS7c) proteins. The red, blue, and orange dotes represent PiDCoV, SpDCoV, and PDCoV strains, respectively. The strains with red front represent the virus that can infect humans.


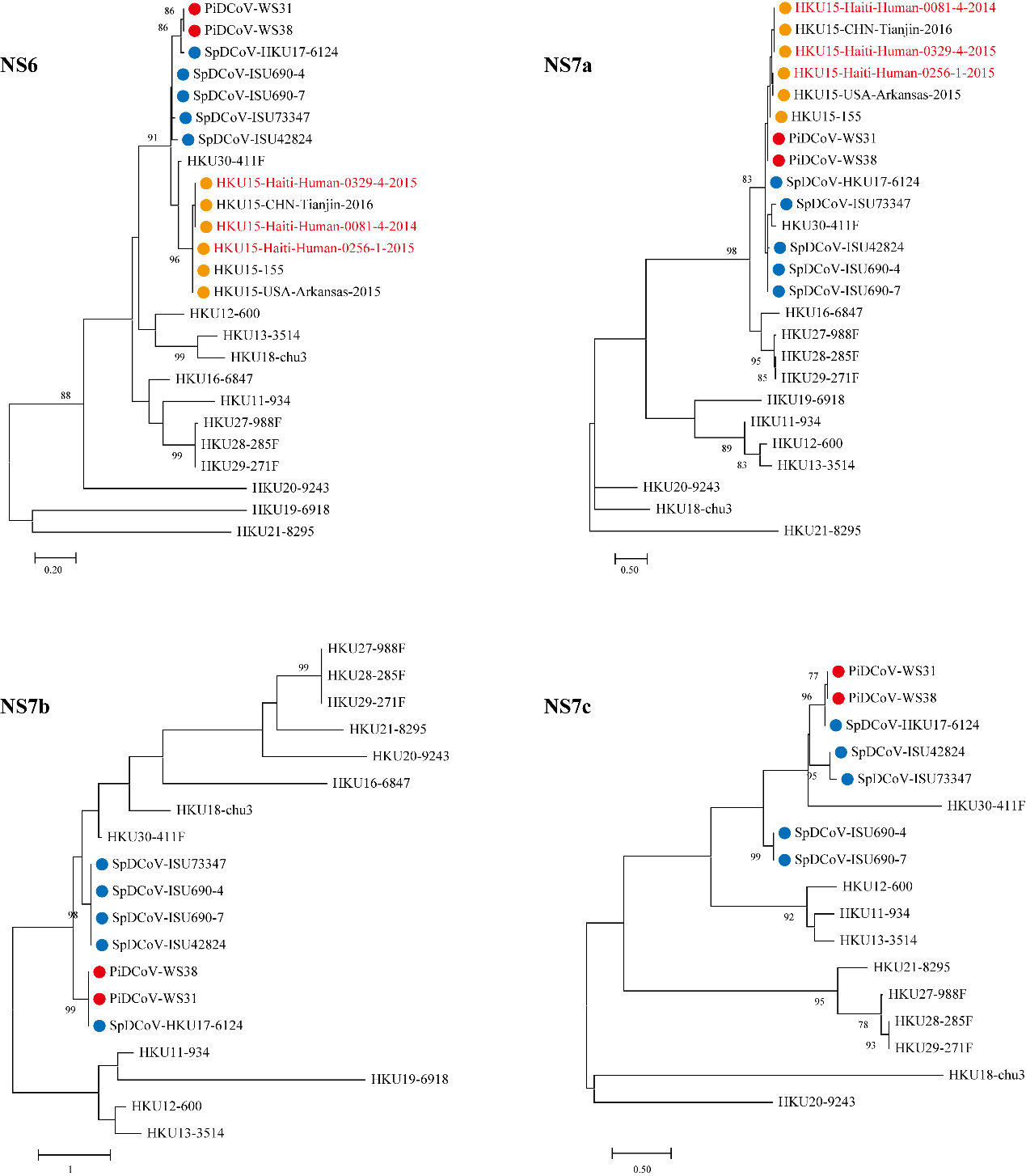

Supplement: Supplemental file 1 — Supplemental material. Download spectrum.00556-23-s0001.docx, DOCX file, 1.9 MB [file spectrum.00556-23-s0001.docx]
